# Improved LC/MS/MS Quantification Using Dual Deuterated Isomers as the Surrogates: A Case Analysis of Enrofloxacin Residue in Aquatic Products

**DOI:** 10.3390/foods12010224

**Published:** 2023-01-03

**Authors:** Yunyu Tang, Guangxin Yang, Essy Kouadio Fodjo, Shouying Wang, Wenlei Zhai, Wenshuai Si, Lian Xia, Cong Kong

**Affiliations:** 1 Key Laboratory of Control of Quality and Safety for Aquatic Products, Ministry of Agriculture and Rural Affairs, East China Sea Fisheries Research Institute, Chinese Academy of Fishery Sciences, Shanghai 200090, China; 2 Laboratory of Constitution and Reaction of Matter, UFR SSMT, Université Felix Houphouet Boigny, 22 BP 582 Abidjan 22, Côte d’Ivoire; 3 Institute for Agri-Food Standards and Testing Technology, Shanghai Academy of Agricultural Sciences, Shanghai 201403, China; 4 Institute of Quality Standard and Testing Technology, Beijing Academy of Agriculture and Forestry Science, Beijing 100097, China; 5 School of Chemistry and Chemical Engineering, Qufu Normal University, Qufu 273165, China

**Keywords:** enrofloxacin, dual isotope surrogates, enrofloxacin-*d_3_*, contaminant detection, accurate quantification, aquatic animals

## Abstract

Extensive and high residue variations in enrofloxacin (ENR) exist in different aquatic products. A novel quantitative method for measuring ENR using high-performance liquid chromatography–tandem mass spectrometry was developed employing enrofloxacin-*d_5_* (ENR-*d_5_*) and enrofloxacin-*d_3_* (ENR-*d_3_*) as isotope surrogates. This reduced the deviation of detected values, which results from the overpass of the linear range and/or the large difference in the residue between the isotope standard and ENR, from the actual content. Furthermore, high residue levels of ENR can be directly diluted and re-calibrated by the corresponding curve with the addition of high levels of another internal surrogate without repeated sample preparation, avoiding the overflow of the instrument response. The validation results demonstrated that the method can simultaneously determine ENR residues from MQL (2 µg/kg) to 5000 × MQL (method quantification limit) with recoveries between 97.1 and 106%, and intra-precision of no more than 2.14%. This method realized a wide linear calibration range with dual deuterated isomers, which has not been previously reported in the literature. The developed method was successfully applied to the analysis of ENR in different aquatic products, with ENR residue levels varying from 108 to 4340 μg/kg and an interval of precision in the range of 0.175~6.72%. These results demonstrate that batch samples with a high variation in ENR residues (over the linear range with a single isotope standard) can be detected by the dual isotope surrogates method in a single sample preparation process.

## 1. Introduction

Enrofloxacin (ENR) is a broad-spectrum antibiotic found in animals as a second-generation fluorinated quinolone. Because of its efficaciousness against common bacterial pathogens, ENR is applied in treating and preventing various bacterial diseases [1], including furunculosis, vibriosis, and bacterial kidney diseases in aquaculture [2,3,4]. At present, ENR is licensed for use at levels below maximum residue limits (MRL), set at 100 µg/kg for ENR and ciprofloxacin (CIP) in fish farming in China, the European Union, and Vietnam [5]. In the United States, no fluorinated quinolone has been approved for use in food-producing animals (except in poultry) since 1997 [6]. Despite this constraint, ENR is extensively applied beyond the set limit value to control diseases in aquaculture, cattle, pigs, and poultry farms [7]. Recent reports have shown high detection rates of 11.4% and 50.4% of ENR residues in aquatic products in South Korea [8] and China [9], respectively. Residues range between N.D. and 785 μg/kg. This excessive use of ENR is the result of over-exploited domestic fisheries, with intensive and high-density culture being adopted to obtain high yields and profits. These activities lead to high antibiotic residues in treated aquatic animals, aquaculture-related sediments and soils, and natural water environments [10,11,12]. This, in turn, leads to potential exposure of human health to risks induced by the ultimate accumulation of ENR in humans and the alarmingly high issues related to antibiotic bacterial resistance [13]. Growing concerns about quality and safety necessitate the monitoring of ENR residues in aquatic products for the safety of human consumption.

The methods for quantitative determination of ENR in aquaculture have been extensively reported. Rapid detection with competitive indirect enzyme-linked immunosorbent assay (ELISA) was performed in field tests due to its easy operation and rapid analysis [14,15]. Furthermore, several studies reported the quantitation of ENR using high-performance liquid chromatography (HPLC) with fluorescence detectors [16,17]. On the other hand, high-performance liquid chromatography–tandem mass spectrometry (HPLC−MS/MS) was also extensively used for the determination of ENR in recent years [18,19]. Because of its high specificity and sensitivity, LC–MS/MS attracted much attention in various fields when applied to the analysis of antibiotics [20,21,22,23]. Generally, the pretreatment of ENR includes solvent extraction, followed by purification with liquid–liquid extraction or solid-phase extraction, concentration, and redissolution before analysis using LC–MS/MS assays. In most cases, the purification and concentration processes can cause the loss of the target analyte, resulting in decreased sensitivity in the detection method.

**Figure 1:**
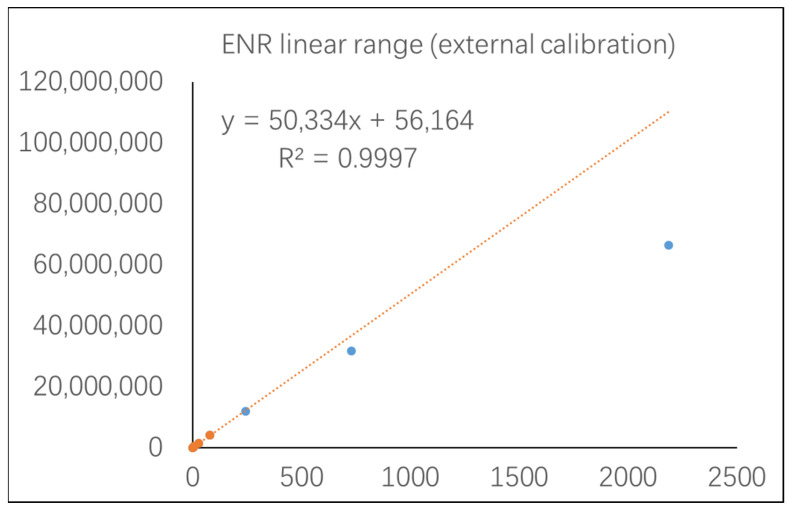
Linear range profile of ENR in the MS detector.

In order to solve this issue, and to obtain more accurate results, an isotope surrogate was introduced as the control of stochastic and/or systematic variation in analyte extraction and analysis [24,25]. Furthermore, the inaccuracy resulting from matrix effects and sample preparation can be effectively compensated by the addition of a fixed amount of isotope surrogate to each sample at the beginning of the process [26]. Therefore, ENR was extensively estimated using the isotope standard method with HPLC–MS/MS in most cases to obtain an accurate measurement. In previous reports, samples containing ENR with concentrations significantly above MRL, such as 785 μg/kg in loach [8], 148.4 μg/kg in carp [27], and 2200 μg/kg in grass carp [28], were found. These high residue values can be attributed to the overapplication of this antibiotic. However, the MS detector tends to produce unstable and imprecise results with the regular detection method, which might be caused by the range of the calibration curve, a high discrepancy between the analyte and the isotope surrogate, and/or the saturated response on the instrument [29]. According to our experience, the linear response range of ENR in the MS detector was not over 500 ng/mL in an actual sample test (Figure 1), as can be found in the recent literature [30,31]. Obviously, the response values of ENR at concentrations of more than 500 ng/mL do not follow the linear calibration curve and tend to be saturated (Figure 1).

Thus, ENR determination requies a repeated sample preparation to obtain more accurate results. It is noteworthy that the dilution of the original sample can reduce the response of MS to resolve the difficulty in MS saturation of the instrument, but the measured value of the diluted sample is still beyond the range of the calibration curve using the quantitative method with the isotope standard [32]. Moreover, the dilution of injection samples cannot resolve the enormous discrepancy in concentration between the analyte and the isotope surrogate to obtain stable and precise results. Therefore, the samples need to be further analyzed by changing the amount of added isotope surrogate and establishing a new procedure, as follows: (i) a new calibration curve is prepared to match the sample concentration; (ii) a repeated sample preparation should be performed with a reasonable amount of added isotope surrogate [33]. However, the high amount of ENR may saturate the MS system detector, resulting in no suitable calibration curve. Consequently, the sample requires laborious re-preparation to resolve the linearity range of the calibration curve, saturation of the MS detector, and the vast discrepancy in concentration between the analyte and the isotope surrogate; this, therefore, increases the time spent on analysis and generates more hazards for the environment. Moreover, the repeatability and reproducibility of the determined results could be poor if a lower amount of isotope surrogate is used than the target ENR in the final solution to be analyzed [34]. This could occur even though the detection values fall within the range of the new calibration curve and the linear response range of the instrument. Therefore, it is necessary to develop a simple, fast, and reliable quantitative method for the analysis of ENR residue levels, which vary from low to high amount in aquatic animals. The use of two different levels of isotope surrogates can provide two ranges of calibration curves, which effectively extends the upper limit of the curve and leads to accurately quantitate the analyte at low and high concentrations, respectively. Moreover, the high addition level of the isotope surrogate can be diluted directly with solutions to adapt the instrument’s response. 

This detection strategy for use with an HPLC–MS instrument has not been proposed and validated in the previous literature. In this study, therefore, a new isotope standard, ENR-*d_3_*, for ENR was synthesized and characterized by ^1^HNMR and MS. We firstly established and validated a novel quantitative method for ENR in aquatic animals using two isotope surrogates with HPLC–MS/MS, achieving a wide range in the calibration curve, single-time sample preparation, direct dilution for the instrument limit, and accurate quantification from low to high residue levels. Moreover, the method involves easy sample preparation, high sensitivity, and a low amount of reagent. Finally, the new method was applied to the determination of ENR in actual positive samples for various aquatic species.

## 2. Materials and Methods

### 2.1. Experimental Materials

All reagents and chemicals used in this study were of analytical or chromatography grade. The enrofloxacin and enrofloxacin-*d_5_* standards (>95% purity) were obtained from Dr. Ehrenstorfer (Augsburg, Germany). The ciprofloxacin (CIP) and 1-bromoethane-2,2,2-*d_3_* were purchased from Shanghai AcmecBiochemical Co., Ltd. (Shanghai, China). N,N-dimethylformamide (DMF) and triethylamine (Et_3_N) were of analytical grade and obtained from Sinopharm Chemical Reagent Co., Ltd. (Shanghai, China). Methanol (MeOH), acetonitrile (MeCN), formic acid, ethyl acetate (EA), dichloromethane, acetic acid, and ammonium acetate of HPLC-grade were from Merck (Darmstadt, Germany). The certified reference material (CRM) (GBW 10167, Batch No. 1672107) was purchased from the Institute of Quality Standards and Testing Products for Agro-products of CAAS. The aquatic product samples were obtained from the local farmers’ market in Shanghai, China. Ultrapure water (18.2 MΩ) was prepared in a Milli-Q water purification system (Millipore Co., Bedford, MA, USA).

### 2.2. Synthesis of ENR-d_3_

**Figure 2:**
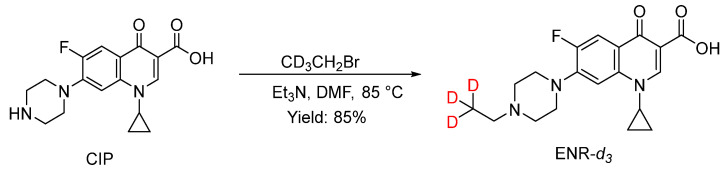
Synthetic route for the target compound.

The reaction was performed according to the synthesis route in Figure 2, which was optimized by a reported method [35]. To a solution of CIP (1.80 g, 5.4 mmol) in DMF (50 mL), Et_3_N (1.20 g, 11.9 mmol) and 1-bromoethane-2,2,2-*d_3_* (0.78 g, 7.0 mmol) were added at room temperature. The reaction mixture was heated to 80 ℃ and further stirred for 2 h under an N_2_ atmosphere. After cooling to room temperature, DMF was removed by a rotatory evaporator under reduced pressure at ca. 80 °C for about 30 min [36], and the residue was purified on a silica gel column using CH_2_Cl_2_:EA = 20:1 as the eluent, yielding the desired product (1.66 g, 85%) as a yellow solid. ^1^H NMR spectra were obtained using a Bruker AM 400 MHz spectrometer (Bruker, Massachusetts, USA) at 298 K using tetramethylsilane (TMS) as the internal standard. HRMS measurements were performed using a Q-Orbitrap mass spectrometer (Q-exactive, Thermo Fisher, USA). ^1^H NMR (400 MHz, D_2_O): δ 8.47 (s, 1H), 7.86 (d, J = 8.8 Hz, 1H), 7.60 (d, J = 4.8 Hz, 1H), 3.65–3.59 (m, 1 H), 3.38–3.24 (m, 4H), 2.82–2.66 (m, 4H), 2.51–2.46 (m, 8H), 1.35–1.29 (m, 2H), 1.15–1.09 (m, 2H), 0.99–0.96 (m, 9H). HRMS (ESI, m/z): calcd for C_19_H_20_D_3_FN_3_O_3_ ([M + H]^+^), 363.1912; found, 363.1887. 

The purity of ENR-*d_3_* was determined by LC–UV [37]. The LC–UV equipment consisted of an Agilent 1100 series with an autosampler injector (Agilent Technologies Inc., State of California, USA). UV detection was performed by a diode array detector (DAD) at a wavelength of 280 nm for ENR-*d_3_*. The ENR was separated by chromatography on a Zorbax Eclipse XDB-C18 (150 mm × 4.6 mm i.d.; Agilent Technologies). 

### 2.3. Sample Preparation

Stock solutions were prepared in methanol at 1 µg/mL for ENR and ENR-*d_5_*, and 100 µg/mL for ENR-*d_3_*. Further dilution was applied for recovery and calibration solutions. The blank samples were prepared using the muscle of grass carp obtained from a local supermarket in Shanghai (China). All blank samples were firstly screened to ensure that they were free of the antibiotics of interest. Two calibration curves (Curve 1 and 2) were established using a standard solution of different concentrations prepared with a blank matrix solution; the curves were fitted by linear regression with ENR-*d_5_* and ENR-*d_3_*, respectively. For the plot of calibration Curve 1, the standard solutions were at 1, 3, 9, 27, 81, and 243 ng/mL of ENR, with 5 ng/mL of ENR-*d_5_* as the isotope standard for each point. For the plot of calibration Curve 2, the standard solutions were 27, 81, 243, 729, 2187, and 6561 ng/mL of ENR with 100 ng/mL of ENR-*d_3_*. Considering that a high amount of ENR can saturate the MS system detector, the standard solutions of Curve 2 can be diluted 20 times to obtain good linearity for the subsequent quantification of the correspondingly diluted sample solution. Finally, the actual concentrations of Curve 2 were 1.35, 4.05, 12.2, 36.4, 109, and 328 ng/mL of ENR with 5 ng/mL of ENR-*d_3_* for determination by LC–MS/MS.

The analyte was extracted following the optimized method in [29]. Briefly, a 5 g homogenized sample was weighed in a 50 mL centrifuge tube and added to 50 ng of ENR-*d_5_* (50 μL of solution of 1 μg/mL ENR-*d_5_* in methanol) and 1000 ng of ENR-*d_3_* (100 μL of solution of 10 μg/mL ENR-*d_5_* in methanol), respectively. An amount of 10 mL of acetonitrile–water solution (85:15, containing 1 mL/100 mL acetic acid) was then added, and the sample was vortex–mixed for 10 min. Afterward, the sample was extracted by ultrasonication for 10 min, and centrifuged (5000× *g*) for 5 min at 4 °C. Following this, a 1 mL aliquot was filtered through a 0.22 μm hydrophobic polytetrafluoroethylene (PTFE) membrane as solution 1 for analysis. Meanwhile, 50 μL of solution 1 was diluted with 950 μL of extraction solvent, and the obtained solution (solution 2) was used for analysis. 

### 2.4. LC–MS/MS Analysis

A HPLC (LC-20A, Shimadzu Corporation, Japan) coupled with a Sciex Qtrap 5500 tandem quadrupole mass spectrometer (Danaher Corporation, Washington, DC, USA) with an electrospray ionization (ESI) source was used for the detection of ENR in the multiple reaction monitoring (MRM) mode. Chromatographic separation was performed on a C18 column (Agilent poreshell 120 EC-C18, 100 × 4.6 mm i.d., 2.7 μm, Agilent Technologies, Santa Clara, CA, USA) under a flow rate of 0.8 mL/min at 40 °C, with an injection volume of 2 μL [38]. Mobile phase A water (containing 1 mL/L of formic acid and 2 mmol/L ammonium acetate) and mobile phase B methanol were used for chromatography separation with the following gradient elution procedure: 15% B for 1.0 min, 15% B to 30% B over 2.5 min, 1.0 min at 30% B, ramping to 95% B over 2.0 min, held for 1.7 min at 95% B, followed by a return to 15% B within 0.1 min and 1.2 min at 15% B for re-equilibration. 

The parameters for mass spectrometry include a spray voltage of 4500 V, a CUR pressure of 42 psi, GS1 and GS2 pressures of 55 psi, an ion source temperature at 550 °C, and CAD of the medium. MS/MS parameters for ENR, ENR-*d_5,_* and ENR-*d_3_* are summarized in Table 1. 

### 2.5. Method Validation

The method quantification limit (MQL) of 2 µg/kg was validated according to the Chinese Standard [39]. The calibration curves were prepared by the peak area ratios of ENR to the isotope standard plotted against the concentration. Recovery and accuracy were examined by comparing the measured concentrations from processed samples with different spiking levels. Intra- and inter-assay precision was assessed by relative standard deviation (RSD) of the measured positive samples. The RSD was calculated for all determinations in a spiking experiment with three replicates. The content of ENR in the samples was calculated according to the following equation:

$$X=\frac{C\times V\times 1000}{m\times 1000}$$

where *X* (µg/kg) is the content of ENR in the samples; *C* (ng/mL) indicates the concentration of ENR detected in the samples; *V* (mL) is the volume of the extraction reagent; and *m* (g) stands for the weight of the sample. 

The decision limit (CCα) and the detection capability (CCβ) of the method were obtained according to Commission Decision 2002/657/EC. We performed the measurement of noise on 20 blank samples (grass carp), with ENR-*d_5_* as the surrogate, and then the decision limit was obtained; this was greater than 3 times the signal-to-noise ratio from the blank sample. Furthermore, we spiked ENR in 20 blank samples (grass carp) at the concentration of CCα, and calculated the detection capability with the sum of CCα + 1.64 times the SD of within-laboratory reproducibility (n = 20).

## 3. Results and Discussion

### 3.1. ^1^HNMR Spectra of ENR-d_3_

**Figure 3:**
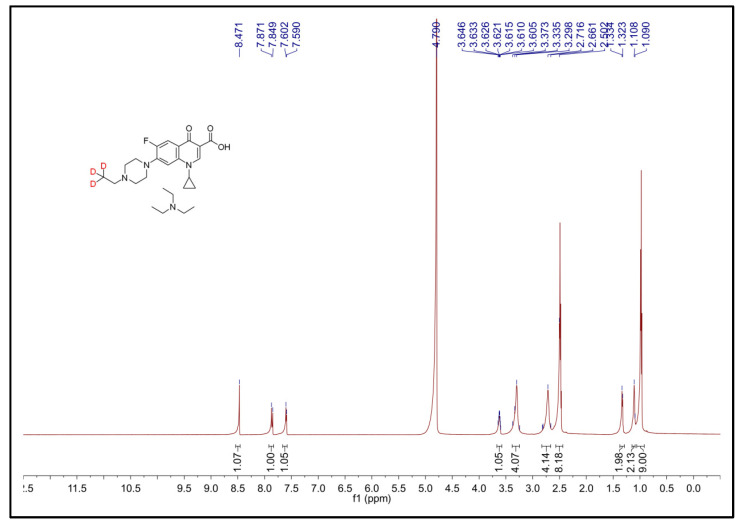
^1^H NMR spectrum of ENR-*d_3_*.

ENR-*d_5_* is commonly used as an isotope standard for ENR. With the aim of obtaining a new stable isotope surrogate, ENR-*d_3_* was synthesized from commercial CIP and 1-bromoethane-2,2,2-*d_3_* via the S_N_2 substitution reaction. The structure of ENR-*d_3_* was firstly characterized by ^1^H NMR and MS analysis. For ^1^H NMR (Figure 3), in comparison with CIP (Figure S1), 15 additional protons were observed; these can be attributed to the Et_3_N salt existing with ENR-*d_3_* in the spectrum. In detail, the two proton signals in methylene linked to the CD_3_ group were overlapped with six methylene proton signals of Et_3_N at 2.51−2.46 ppm. The nine proton signals in the methyl group for Et_3_N were at 0.99−0.96 ppm. All other signals were in good agreement with the original CIP. The m/z 363 in ENR-*d_3_* was obtained from product ion mass spectra (Figure S2), and the m/z 102 at the top of this figure shows the protonated Et_3_N, which was also detected in the ENR-*d_3_* ^1^HNMR spectra (Figure 3). Pure m/z 363 without an m/z 360 signal demonstrated the high purity of the synthesized ENR-*d_3_* without the presence of ENR. Moreover, the HPLC showed that the purity was >95% (Figure S3). Hence, the synthesized ENR-*d_3_* can be applied for HPLC−MS/MS analysis as an isotope surrogate.

### 3.2. Calibration Range and Deviation

**Figure 4:**
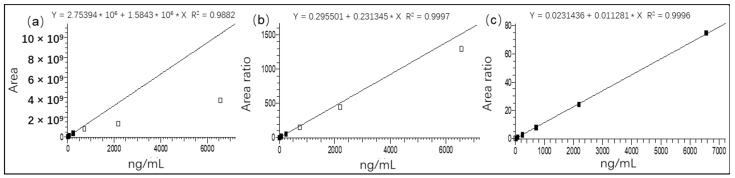
Calibration curves of ENR with the external method (**a**), ENR-d5 (**b**), and ENR-d3 (**c**) for internal calibration.

With the aim of detecting ENR with a high variation in concentration in one batch of samples, two calibration curves were individually prepared using ENR-*d_5_* (5 ng/mL) and ENR-*d_3_* (100 ng/mL) as isotope surrogates with blank matrix solutions. The two isotope surrogates can compensate for extraction loss during the sample pretreatment and instrument measurement [40]. As shown in Figure 4, the calibration curves were tested for external calibration, and with ENR-*d_5_* and ENR-*d_3_* for internal calibration, respectively. It is clear that with such a wide calibration range, the external calibration did not produce a good coefficient of determination (*r^2^* < 0.99), while internal calibration with ENR-*d_5_* and ENR-*d_3_* allowed a satisfactory calibration of *r^2^* > 0.999. The internal calibration curves exhibited linearity with the ratio of the peak area of the analyte/ isotope standard (y) and the concentration of ENR (x). Firstly, the two calibration curves with different isotope surrogates covered a wide linear range of 1−6561 ng/mL. However, the measured concentration deviating from the specified amount was more than 10% above 243 ng/mL in the calibration curve with ENR-*d_5_* (Table S1). On the other hand, the deviations between the spiked and measured values were up to 32% and 111% at concentrations of 1 ng/mL and 3 ng/mL, respectively, in the curve with ENR-*d_3_* (Table S2). The deviations at different concentrations with two isotope surrogates demonstrated the difficulty of accomplishing accuracy when a large concentration difference between the isotope surrogates and the analyte is present. In our study, MQL was set as the first point of the calibration curve using ENR-*d_5_*, varying from 1 to 243 ng/mL (2−486 μg/kg) with a coefficient of determination of 0.9997 (Table 1). For the calibration curve using ENR-*d_3_*, the range for ENR was from 27 to 6561 ng/mL (54−13,122 μg/kg) with *r*^2^ = 0.9996. As a result, the entire calibration curve, covering a wide quantification range from 2 to 13,122 μg/kg, was established for ENR detection. 

### 3.3. Method Validation

**Figure 5:**
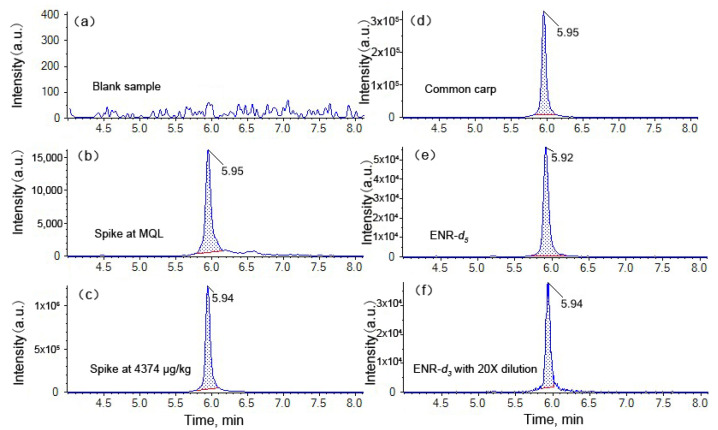
HPLC−MS/MS chromatograms: (**a**) blank sample (EIC of ENR); (**b**) blank samples spiked with ENR at an MQL of 2 µg/kg (EIC of ENR); (**c**) blank samples spiked with ENR at 4374 µg/kg (EIC of ENR); (**d**) positive sample, common carp (EIC of ENR); (**e**) EIC of ENR-*d_5_*; (**f**) EIC of ENR-*d_3_* (samples in (**c**) and (**f**) were diluted at a ratio of 1:20).

For the spiking experiments, the ENR standard was spiked in blank samples at 2, 6, 54, 486, 1458, and 4373 µg/kg, respectively, with the further addition of ENR-*d_5_* (50 ng) and ENR-*d_3_* (1000 ng) as isotope standards. Sample preparation was performed according to the above-mentioned procedure. Afterward, two solutions (solution 1 and solution 2) for each sample were measured by HPLC–MS/MS. Compared with the reported methods, our sample preparation process is obviously easier to operate without any enrichment [1] or clean up using SPE material [41], and/or hexane [42]. Furthermore, the sample can be directly diluted using the dual internal calibration method and then quantified using the isotope surrogate with a high concentration, which is feasible for accurate quantification of high residue samples without repeated sample preparation. Thus, it is time-efficient and requires less reagent and fewer consumables. Notably, linearity of the calibration curves was achieved regardless of whether ENR-*d_5_* was used at low or high levels of concentration. Similar results were observed for ENR-*d_3_*. In other words, the concentrations of ENR-*d_5_* and ENR-*d_3_* can be interchanged with a similar good linearity of the calibration. The spiked samples at 1458 and 4374 µg/kg were quantified using solution 2 with ENR-*d_3_* as the isotope standard. The residue at MQL of 2 μg/kg was obtained with ENR-*d_5,_* and the CCα and CCβ were calculated to be 0.5 μg/kg and 1.5 μg/kg, respectively. As shown in Figure 5a, the blank sample shows a clean background signal with this method, and an excellent sensitivity is obtained (Figure 5b). The spiking experiment at 4374 µg/kg does not show a saturation response on the chromatogram (Figure 5c). Recovery ranging from 97.1% to 106% was observed at all levels of spiking samples (Table S3). Precision values evaluated by the RSD of the measurements of three positive samples in different matrices were found to be below 6.90% and 6.49% for intra- and inter-precision, respectively (Table S4 and S5). These results demonstrate that the new method was efficient during extraction, and fully satisfied the requirements of quantification for a broad ENR residue range. It is difficult to find a fish muscle CRM with high ENR residue levels. However, we conducted a test with our dual isotope surrogate method on a newly obtained CRM. The specified ENR residue level of the CRM was 62.5 ± 6.3 μg/kg. The residue level of this CRM could be quantified both with ENR-*d_5_* and ENR-*d_3_*. By using the dual surrogate method, residue levels of 58.7 ± 2.3 μg/kg and 64.1 ± 1.2 μg/kg were obtained with the calibration curve prepared using ENR-*d_5_* and ENR-*d_3_*, respectively. Moreover, we performed a spiked experiment on this positive sample with a high spiking amount, which was quantified using the dual isotope surrogate method. The result also shows a higher accuracy and stability with ENR-*d_3_* than with ENR-*d_5_* (Table 2). This demonstrates the good accuracy of the developed method. Furthermore, this method was compared with previous methods using mass spectrometry for testing for ENR residues in aquatic products, as shown in Table 3. Our method has comparable sensitivity and stability, but a much wider linear range through the use of dual isotope surrogates, which demonstrates the advantages of our method in the sample test for high ENR residue levels.

### 3.4. Application of Dual Isotope Surrogates to Aquatic Products

The proposed method was applied in the analysis of a batch of different aquatic products collected from commercial markets. A total of 47 out of 136 samples were detected as positive. Four samples were found to exceed the linear range of the calibration curve with ENR-*d_5_* as the surrogate. Three positive samples were then selected for validation of the method; these were a common carp (*Cyprinus carpio*), a bullfrog (*Lithobates catesbeiana (Shaw)*) and a bluntnose black bream (*Megalobrama amblycephala*), which showed a medium, high and significantly high concentration of ENR, respectively.

The ENR content in three aquatic products, determined using dual isotope surrogates, is summarized in Table 4. HPLC–MS/MS analyses show that the new isotope surrogate ENR-*d_3_* was successfully applied and detected in positive aquatic products. As a result, the detection of ENR was achieved using the two isotope surrogates, respectively. A slight difference in the detected concentrations and the RSD values between the two quantitative results was found. As shown in Table 4, ENR contents of 108 ± 7.25, 681 ± 35.7, and 3903 ± 433 μg/kg, using ENR-*d_5_* for quantitation, were detected in the bluntnose black bream, the common carp (Figure 5d), and the bullfrog, respectively. Meanwhile, the RSD values were found to be 6.72, 5.24, and 11.1%, respectively. It should be noted that the SD and RSD values of the common carp and bullfrog were relatively high, suggesting that the higher ENR concentrations were comparable with the added ENR-*d_5_*. This effect results in relatively low repeatability and reproducibility of the measurement. On the other hand, all samples were measured with ENR at levels of 99.1 ± 0.173, 624 ± 4.95, and 4340 ± 21.2 μg/kg using ENR-*d_3_*, respectively, and RSD values ranged from 0.175 to 0.794%. The response of ENR-*d_3_* in solution 2 was comparable with ENR-*d_5_* in solution 1 (Figure 5e,f). Clearly, the values of the two quantitative methods agreed well with each other. Meanwhile, the calculated SD and RSD values were much lower using the dual isotope surrogate method than those quantified by ENR-*d_5_* alone. It is worth noting that the ENR level in bluntnose black bream shows a discrepancy of around 8% between the result with ENR-*d_5_* and ENR-*d_3_*. We assume that both results are suitable for quantitation with both ENR-*d_5_* and ENR-*d_3_*. However, higher SD and RSD values were observed for ENR-*d_5_* as the isotope surrogate. This result may be interpreted by the variation in isotope surrogate loss through the preparation of the sample due to lower amounts of ENR-*d_5_*. Therefore, the addition of more ENR-*d_3_* could improve the stability of quantitation due to the stable synchronous compensation effect for the analyte and isotope surrogate loss during sample preparation. On the other hand, the results of ENR-*d_5_* are not acceptable for formal reports, as they was obtained using a calculation curve where the linear range does not cover this value. The advantage of using dual deuterated isomers was not obvious for the practical sample with only a low ENR residue. It does work and saves time for samples with a high ENR residue, especially when the result with single surrogates exceeds the linear range of the calibration curve. These observations suggest that the accuracy and precision of quantitative results can be achieved by choosing suitable isotope surrogates. Therefore, the experimental results demonstrate that using different levels of dual isotope surrogates could provide accurate and reproducible results in one preparation.

## 4. Conclusions

In summary, ENR-*d_3_* was firstly synthesized as a new isotope surrogate to establish an accurate and fast quantitative method for measuring ENR residues in aquatic products with dual isotope surrogates (ENR-*d_5_* and ENR-*d_3_*) using HPLC–MS/MS. The new method can be used to perform the determination of ENR with a wide linear range from 2 to 13,122 μg/kg in a single sample preparation with excellent sensitivity, accuracy, and precision. Moreover, a high residue level of ENR, which generally leads to the overflow of the instrument response, can be directly diluted and re-calibrated by the corresponding curve with a high addition level of the other internal surrogate without repeating sample preparation. Furthermore, it was also possible to reduce the deviation of the detected value caused by the vast concentration difference between the isotope standard and target analytes. The new method was applied for the determination of ENR in positive samples for various aquatic species showing the ENR values ranging from 99.1 to 4340 μg/kg. The new method was demonstrated to be time-efficient and easy to operate, providing a practical, accurate, and precise way to determine veterinary drug residues. It is especially suitable for batch samples with a wide range of residue variations and possible high residue content.

## Figures and Tables

**Table 1:** MS/MS parameters and standard curves with two isotope surrogates.

Compounds	Precursor Ion (m/z) and Adduct	Product Ions (m/z) and Theoretical Fragments	CE (eV)	Calibration Curves	Linear Range (ng/mL)	*r* ^2^
ENR	360 ([M + H]^+^)	316 * (C_18_H_23_FN_3_O^+^)	19	y = 0.295501 + 0.231345 *× (ENR-*d_5_*)	1–243	0.9997
				y = 0.0231436 + 0.011281 *× (ENR-*d_3_*)	27−6561	0.9996
		245 (C_14_H_14_FN_2_O^+^)	26	−	−	−
ENR-*d_5_*	365 ([M + H]^+^)	321 (C_18_H_17_D_5_FN_3_O^+^)	19	−	−	−
ENR-*d_3_*	363 ([M + H]^+^)	319 (C_18_H_20_D_3_FN_3_O^+^)	19	−	−	−

* Quantitative ion. CE, collision energy; ***r*^2^**, coefficient of determination.

**Table 2:** Recovery and RSDs of a spiking experiment in a positive sample with the dual isotope surrogates method for the determination of ENR (n = 3).

Spike Amount (µg/kg)	ENR-*d_5_* as Internal Surrogate				ENR-*d_3_* as Internal Surrogate			
	Detected in Blank (µg/kg)	Detected After Spiking (µg/kg)	Recovery (%)	RSD (%)	Detected in Blank (µg/kg)	Detected After Spiking (µg/kg)	Recovery (%)	RSD (%)
10,000	58.7	7632	75.7	12.3	64.1	10,207	101	3.81

**Table 3:** Comparison of the developed method with previous reports of ENR residue tests using mass spectrometry.

Calibration Method	Instrument	Linear Range (μg/L)	LOQ (μg/kg)	Stability
Internal calibration	LC–MS/MS	0.2–100	1.0	6.7% (spiked at 2 μg/kg) [9]
External calibration	LC–MS/MS	12.5–75.0	12.5	9.2% (spiked at 25 μg/kg) [29]
External calibration	LC–MS/MS	0.5–600	0.5	3% (spike amount not mentioned) [8]
Internal calibration	LC-Q-Orbitrap MS	5–500	1.0	12.8% (spiked at 1 μg/kg) [1]
Internal calibration	LC–MS/MS	1–6561	2.0	2.14% (spiked at 2 μg/kg) (this study)

**Table 4:** Determination of ENR in three aquatic products using ENR-*d_5_* and ENR-*d_3_* as the isotope standards (n = 9).

Sample Type	ENR-*d_5_* as Isotope Surrogate		ENR-*d_3_* as Isotope Surrogate	
	Concentration ± SD (μg/kg)	RSD (%)	Concentration ± SD (μg/kg)	RSD (%)
Bluntnose black bream	108 ± 7.25	6.72	99.1 ± 0.173	0.175
Common carp	681 ± 35.7	5.24	624 ± 4.95	0.794
Bullfrog	3903 ± 433	11.1	4340 ± 21.2	0.489

## Data Availability

The data presented in this study are available on request from the corresponding author.

## Supplementary Materials

The following supporting information can be downloaded at: https://www.mdpi.com/article/10.3390/foods12010224/s1.
