# Supplementary material for: Improved LC/MS/MS Quantification Using Dual Deuterated Isomers as the Surrogates: A Case Analysis of Enrofloxacin Residue in Aquatic Products
Source: Foods. 2023 Jan 3;12(1):224. doi: 10.3390/foods12010224 (PMC9818688; doi:10.3390/foods12010224)
Supplement: Supplementary file 1 [file foods-12-00224-s001.zip › foods-2033526-supplementary.pdf]

Supplementary Materials for

Improved LC/MS/MS Quantification using Dual Deuterated Isomers as the Surrogates: a Case Analysis of Enrofloxacin Residue in Aquatic Products

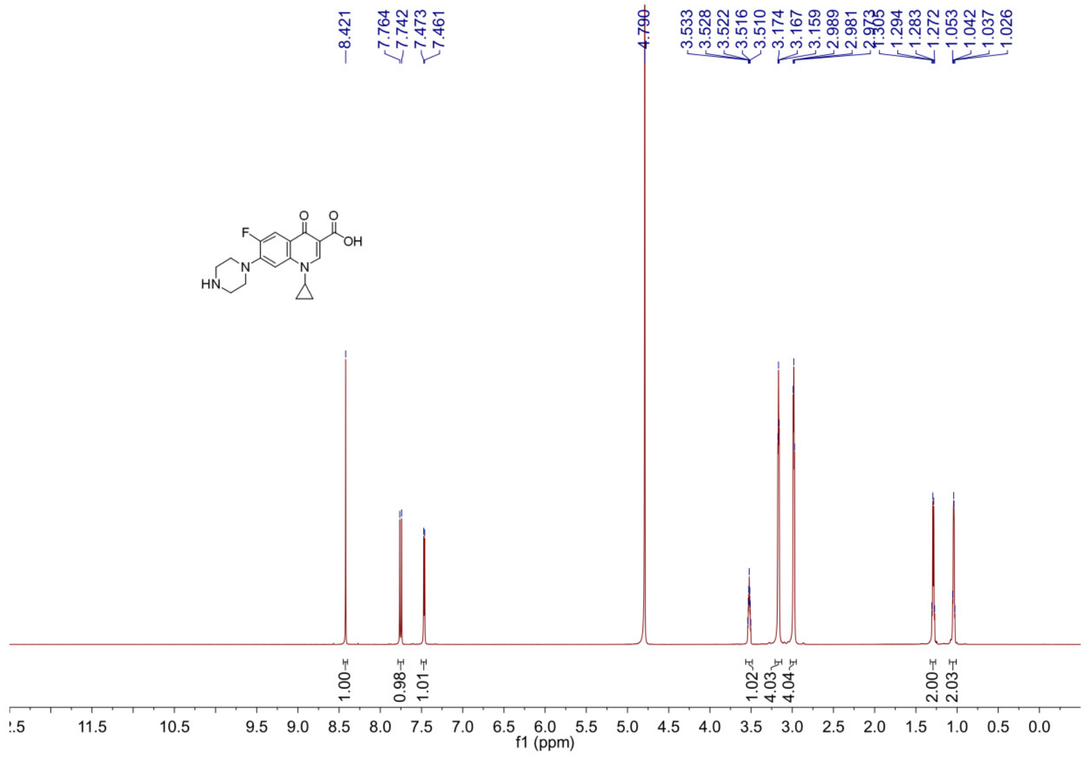

Figure S1. The ^{1}H NMR spectrum of CIP.

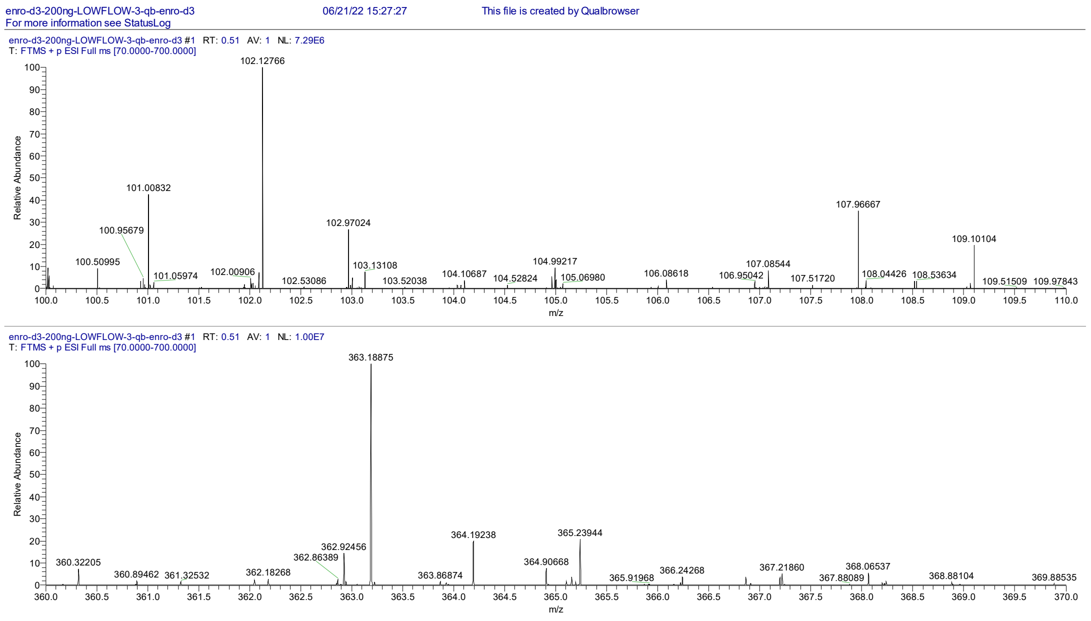

Figure S2. The mass spectra of ENR-d_3.

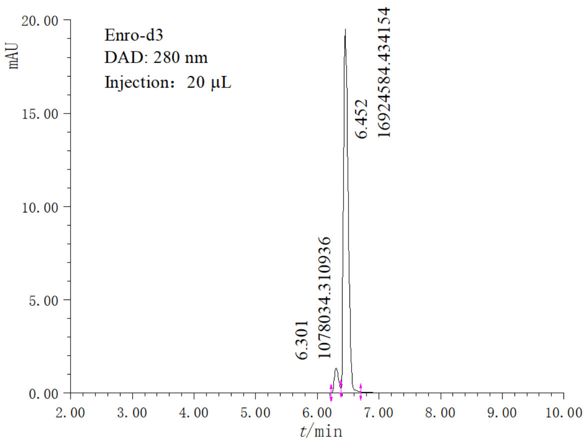

Figure S3. The HPLC chromatogram of ENR-d_3.

Table S1. Standard curve details using ENR-d_5 (5 ng/mL) as isotope surrogate.

Point	Peak area of ENR	Peak area of ENR-d_5	Area Ratio	Specified Amount (ng/mL)	Measured concentration (ng/mL)	Diff (%)	RT (min)
STD1	3425343	6577629	0.521	1.000	0.974	-3%	8.09
STD2	7787517	8226022	0.947	3.000	2.815	-6%	8.09
STD3	20718550	8396876	2.467	9.000	9.388	4%	8.08
STD4	55365279	8135286	6.806	27.000	28.140	4%	8.08
STD5	142804954	7413661	19.262	81.000	81.985	1%	8.08
STD6	363111558	6486488	55.980	243.000	240.698	-1%	8.08
STD7	794777344	5248702	151.424	729.000	653.259	-10%	8.07
STD8	1338159958	2979893	449.063	2187.000	1939.821	-11%	8.08
STD9	3670221311	2817502	1302.651	6561.000	5629.498	-	8.06

							14%

Table S2. Standard curve detail using ENR-d_3 (100 ng/mL) as isotope surrogate.

Point	Peak area of ENR	Peak area of ENR-d_3	Area Ratio	Specified Amount (ng/mL)	Measured concentration (ng/mL)	Diff (%)	RT (min)
STD1	186391	8605003	0.022	1.000	-0.114	-111%	8.09
STD2	427814	9343252	0.046	3.000	2.053	-32%	8.09
STD3	1165056	9593107	0.121	9.000	8.696	-3%	8.08
STD4	3209888	9622126	0.334	27.000	27.514	2%	8.08
STD5	7685495	8045451	0.954	81.000	82.471	2%	8.08
STD6	20687227	7456519	2.774	243.000	243.841	0%	8.08
STD7	48504399	6018656	8.059	729.000	712.337	-2%	8.07
STD8	78913115	3293850	23.958	2187.000	2121.677	-3%	8.08
STD9	254776662	3400132	74.931	6561.000	6640.159	1%	8.06

Table S3. Spiked recovery of the dual isotope surrogates method for the determination of ENR (n = 3)

Surrogate	Added (μg/kg)	Analyte Area	Surrogate area	Area Ratio	Measured value (ng/mL)	Detected amount (μg/kg)	Average amount (μg/kg)	Recovery (%)	RSD (%)
ENR-d_5	2	3332454	7386770	0.451	1.02	2.05	2.05	102	2.14
		3115693	6986770	0.446	1.00	2.00			
		3736471	8186770	0.456	1.05	2.09			
	6	6272492	6575900	0.954	3.13	6.27	6.36	106	1.33
		6011838	6176550	0.973	3.22	6.43			
		5778265	5968448	0.968	3.19	6.39			
	54	43800039	6454636	6.786	27.62	55.23	55.75	103	0.96
		42032617	6143255	6.842	27.85	55.70			
		41400937	5988336	6.914	28.15	56.30			
	486	320021068	5647782	56.663	236.99	473.97	475.34	97.8	0.25
		305015547	5361915	56.886	237.92	475.84			
		304500884	5348613	56.931	238.11	476.22			
ENR-d_3	1458	33910510	4215195	8.045	717.19	1434.39	1429.45	98.0	0.39
		31393494	3932133	7.984	711.73	1423.45			
		34569835	4308705	8.023	715.26	1430.52			
	4374	103966843	4374326	23.768	2126.66	4253.32	4248.93	97.1	0.39
		99696957	4217224	23.640	2115.27	4230.53			
		104276114	4377460	23.821	2131.47	4262.93			

Table S4. Positive samples and precision testing for the determination of ENR with ENR-d_5

Sample type	Measured content (μg/kg)	Intra-Mean concentration (μg/kg)	Intra-RSD (%)	Inter-RSD (%)	Mean concentration (μg/kg)	RSD (%)
Common carp	733<br>666<br>728	709 ± 37.3	5.26	5.14	681 ± 35.7	5.24
	695<br>695<br>689	693 ± 3.46	0.450			
	647<br>638<br>641	642 ± 4.58	0.713			
Bull Frog	4367<br>4553<br>3047	3989 ± 821	20.6	3.91	3903 ± 433	11.1
	4031<br>3936<br>4011	3993 ± 50.1	1.25			
	3660<br>3776<br>3744	3727 ± 59.9	1.61			
Bluntnose black bream	109<br>112<br>124	115 ± 7.93	6.90	6.49	108 ±7.25	6.72
	107<br>108<br>108	108 ± 0.577	0.536			
	101<br>101<br>101	101 ± 0.00	0.00			

Table S5. Positive samples and precision testing for determination of ENR with ENR-d_3

Sample type	measured content (μg/kg)	Intra-Mean concentration (μg/kg)	Intra-RSD (%)	Inter-RSD (%)	Mean concentration (μg/kg)	RSD (%)
Common carp	621					
	620	620 ± 0.577	0.0931			
	620					
	635					
	626	629 ± 4.93	0.784	0.791	624 ± 4.95	0.794
	627					
	621					
	621	621 ± 0.577	0.0929			
	622					
Bullfrog	4300					
	4353	4333 ± 29.0	0.670			
	4347					
	4354					
	4340	4346 ± 7.09	0.163	0.150	4340 ± 21.2	0.489
	4345					
	4357					
	4308	4340 ± 28.0	0.645			
	4356					
Bluntnose black bream	99.2					
	98.9	99.0 ± 0.153	0.154			
	99.0					
	99.3					
	99.1	99.2 ± 0.100	0.101	0.121	99.1 ± 0.173	0.175
	99.2					
	99.2					
	98.9	99.0 ± 0.208	0.210			
	98.8					
